# Single-cell-led drug repurposing for Alzheimer’s disease

**DOI:** 10.1038/s41598-023-27420-x

**Published:** 2023-01-05

**Authors:** Silvia Parolo, Federica Mariotti, Pranami Bora, Lucia Carboni, Enrico Domenici

**Affiliations:** 1 Fondazione the Microsoft Research-University of Trento Centre for Computational and Systems Biology (COSBI), 38068 Rovereto, Italy; 2 Department of Pharmacy and Biotechnology, Alma Mater Studiorum University of Bologna, 40126 Bologna, Italy; 3 Department of Cellular, Computational and Integrative Biology (CIBIO), University of Trento, 38123 Trento, Italy

**Keywords:** Data integration, Gene regulatory networks, Network topology

## Abstract

Alzheimer’s disease is the most common form of dementia. Notwithstanding the huge investments in drug development, only one disease-modifying treatment has been recently approved. Here we present a single-cell-led systems biology pipeline for the identification of drug repurposing candidates. Using single-cell RNA sequencing data of brain tissues from patients with Alzheimer’s disease, genome-wide association study results, and multiple gene annotation resources, we built a multi-cellular Alzheimer’s disease molecular network that we leveraged for gaining cell-specific insights into Alzheimer’s disease pathophysiology and for the identification of drug repurposing candidates. Our computational approach pointed out 54 candidate drugs, mainly targeting MAPK and IGF1R signaling pathways, which could be further evaluated for their potential as Alzheimer’s disease therapy.

## Introduction

Alzheimer’s disease (AD) is an age-related neurodegenerative disorder that slowly destroys memory and thinking skills. It is characterized by long preclinical and prodromal phases and an average clinical duration of 8–10 years^1^. According to estimates by the US Alzheimer's Association, 6.5 million Americans aged 65 and older are living with AD in 2022 (https://www.alz.org/).

Genetically, two forms of AD can be distinguished: a familial early-onset AD caused by rare mutations with high effect size and a late-onset sporadic AD caused by a combination of many genetic risk variants with small effect size and environmental factors^2^. The strongest genetic risk factor for late-onset AD is the apolipoprotein E ε4 allele (APOE4), which explains a large fraction of the estimated disease heritability^3^. In addition, genome-wide association studies (GWASs) have identified numerous risk loci associated with late-onset AD^2,4^. Despite this progress in understanding the genetic architecture of AD, the functional contribution of the risk loci to the disease pathophysiology is still mostly unknown^5^ and this hampers the identification of new drug targets and the development of new treatments.

Pathologically, the hallmarks of AD are the accumulation of extracellular amyloid-β plaques and intracellular neurofibrillary tangles composed of aggregated protein tau^6,7^. These toxic aggregates co-occur with other pathological processes such as neuroinflammation, cerebrovascular deregulation, ion channel dysfunction, mitochondrial dysfunction, and oxidative stress that, in turn, lead to synapse and neuronal loss^8^.

In the past twenty years, huge efforts have been devoted to the development of treatments that could modify the disease mechanisms, mainly focusing on amyloid and tau accumulation^9^. Only recently Aduhelm (aducanumab) reached the market as the first disease-modifying treatment for AD, approved by the US Food and Drug Administration (FDA) using the accelerated approval pathway in June 2021. Aducanumab is a monoclonal antibody that was shown to reduce the amyloid plaque burden in the brain in a dose- and time-dependent fashion in two phase 3 clinical trials. Despite this evidence, the primary clinical endpoint, a reduction in cognitive decline, was reached only by one of the two clinical studies and a post-approval clinical trial will be needed to further evaluate the clinical benefits of the drug^10^. Given the multifactorial nature of the disease, it is also reasonable to think that combination therapy would be needed to counteract the disease processes^11,12^. For these reasons, new efforts for the development of AD treatments are urgent.

An effective strategy to reduce time, safety concerns, and cost of drug development is drug repurposing (or repositioning), which involves the investigation of existing drugs for new therapeutic purposes. Numerous computational methods for drug repurposing have been developed^13^. Among them, network-based approaches emerged as an efficient way to integrate heterogeneous layers of information^14,15^ and predict repurposing candidates^16^. We previously developed a network-based drug-disease proximity score to identify drug repurposing candidates^17^. The score was originally applied to tissue-specific networks for the identification of drugs that could be repurposed for metabolic syndrome. Here we aim at extending the applicability of the score to cell-specific networks. Thanks to single-cell RNA sequencing technology, it is now possible to study at the single-cell level the different cell populations constituting a tissue and infer their cell-to-cell communication^18,19^. This enables to gain a cell-specific mechanistic understanding of the biological systems and identify new drug targets^20^.

To characterize the molecular basis of AD and identify drugs that could be repurposed for AD, we developed a cell-specific systems biology workflow. By integrating several layers of information, we built a multi-cellular AD network that we leveraged for the identification of actional targets and drugs that could be further investigated as potential therapeutic opportunities for AD.

## Results

### Overview of the computational pipeline

**Figure 1:**
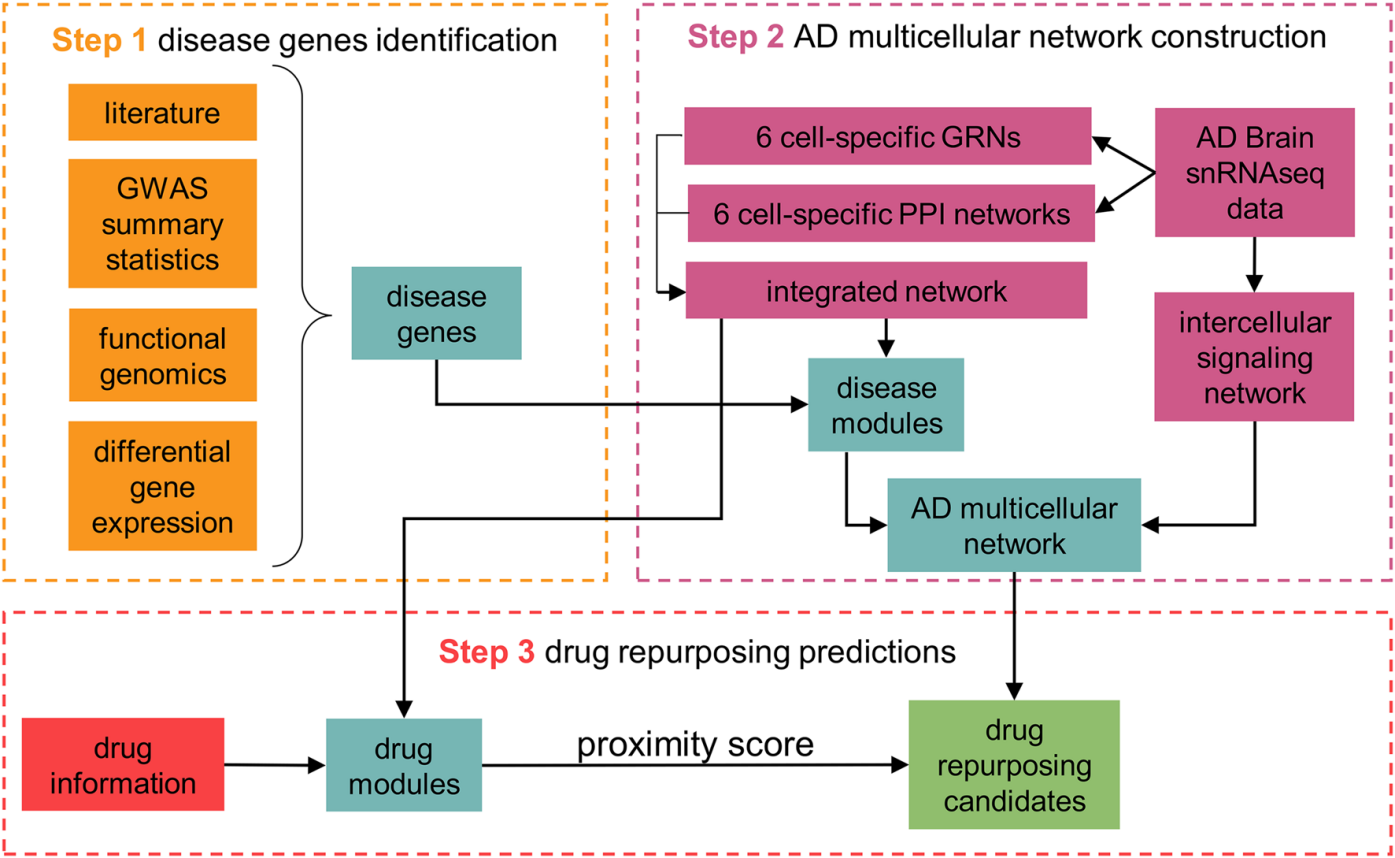
Outline of the computational pipeline. Each dashed line box corresponds to a main step of the pipeline. Step 1 takes as input different data sources and merge them to define six lists of cell-specific disease genes; step 2 builds the AD multicellular network starting from TF-gene interaction and protein–protein interactions; step 3 takes as input the results of step 1 and 2 to identify drug repurposing candidates by means of network analysis.

The computational pipeline we developed aims to gain functional insights into AD and identify drug repurposing candidates by leveraging publicly available single-nucleus RNA-seq (snRNA-seq) data obtained from the prefrontal cortex of post-mortem AD subjects^21^ that we re-analyzed using a network-based approach. The analysis included six cell types: excitatory neurons (EX), inhibitory neurons (IN), microglia (MIC), astrocytes (AST), oligodendrocytes (OLI), and oligodendrocyte progenitor cells (OPC). The pipeline we followed is composed of three main steps: (1) identification of genes associated with AD, (2) construction of a disease-specific multi-cellular network, and (3) network-based identification of drug repurposing candidates (Fig. 1). In the first step, the disease genes have been identified using a multi-pronged approach that includes cell-specific information such as the gene expression levels in the brain cells and the cell-level differential gene expression between AD subjects and controls. This cell-type-aware disease characterization was used in the second step as a basis to define cell-specific disease subnetworks (AD modules), i.e., portions of each cell network enriched in disease genes. We then build a multi-cellular AD network connecting AD modules by leveraging the cell-to-cell signaling that we inferred through ligand-receptor analysis. In step 3, approved drugs with a known protein target have been tested for being AD repurposing candidates using the network-based drug-disease proximity score we previously developed^17^.

### Identification of AD genes

To define a set of AD-associated genes (AD genes), we considered three sources of evidence: genetics, gene expression, and literature. To obtain genetic and gene expression evidence, we focused on the results of AD GWAS and single nucleus RNA-seq (snRNA-seq) of AD brains and controls. These two approaches have been chosen because they give unbiased, high-throughput insight into disease mechanisms that in the case of snRNA-seq reaches single-cell resolution. These sources were complemented with literature data that may capture the results of targeted approaches focused on candidate disease pathways.

The genetic evidence was derived from the summary statistics of the two largest GWAS of AD^22,23^ at the time of the analysis. This data was analyzed to identify AD genes using both genomic proximity and brain expression quantitative trait loci (eQTL) information and combined with publicly available cell-specific functional genomic annotations. To convert the SNP-level associations to gene-level associations using SNP-gene genomic proximity we applied MAGMA^24^, instead to map the SNPs to genes based on tissue-specific eQTL information we applied E-MAGMA^25^ (see details in Methods section). Functional genomics information were obtained from the results of the study on noncoding regulatory regions in brain cells published by Nott et al.^26^. From this study, we identified 57 genes whose expression in neurons (both excitatory and inhibitory) is affected by AD risk variants located in promoters and enhancers. From the same study we also retrieved 41 genes with evidence of modulated expression in microglia due to AD-associated regulatory SNPs and 25 genes for oligodendrocytes. Additional functional annotations from publicly available databases were exploited to annotate the disease genes identified by genomic proximity, as described below. Moreover, since the GWAS loci typically include several genes not all involved in the disease pathophysiology, the GWAS-derived gene-level associations without any functional evidence were not considered as AD genes.

For the gene expression evidence, cell-specific differentially expressed genes were obtained from Mathys et al.^21^ that compared gene expression levels of six brain cell types obtained from AD and control subjects using the same snRNA-seq data we leveraged to build the networks.

Literature evidence was derived from four different sources: Harmonizome^27^, Agorà (https://agora.ampadportal.org/), Kegg^28^, and the Alzheimer's Gene Ontology annotation Aruk (https://www.ebi.ac.uk/GOA/ARUK). We selected these resources because they are recent and already provide an integration on numerous datasets, providing a comprehensive picture of known AD genes. Since the dataset of differentially expressed genes and the functional genomics annotations are cell-specific, the list of disease genes differs across the considered cells and thus we obtained six lists of AD genes, one for each cell type.

Overall, we identified 2653 AD genes, expressed in at least one of the considered brain cells (Supplementary Fig. 1 and Supplementary Table 1). The identified disease genes have been ranked by defining a score that sums the sources of evidence and adds a diversification term according to the number of sources supporting the association (see Methods). Three genes from the EX network (*BIN1*, *CISD1*, and *IL34*) and two genes from the MIC network (*APOE* and *APOC1*) were found in all the three evidence source categories.

**Figure 2:**
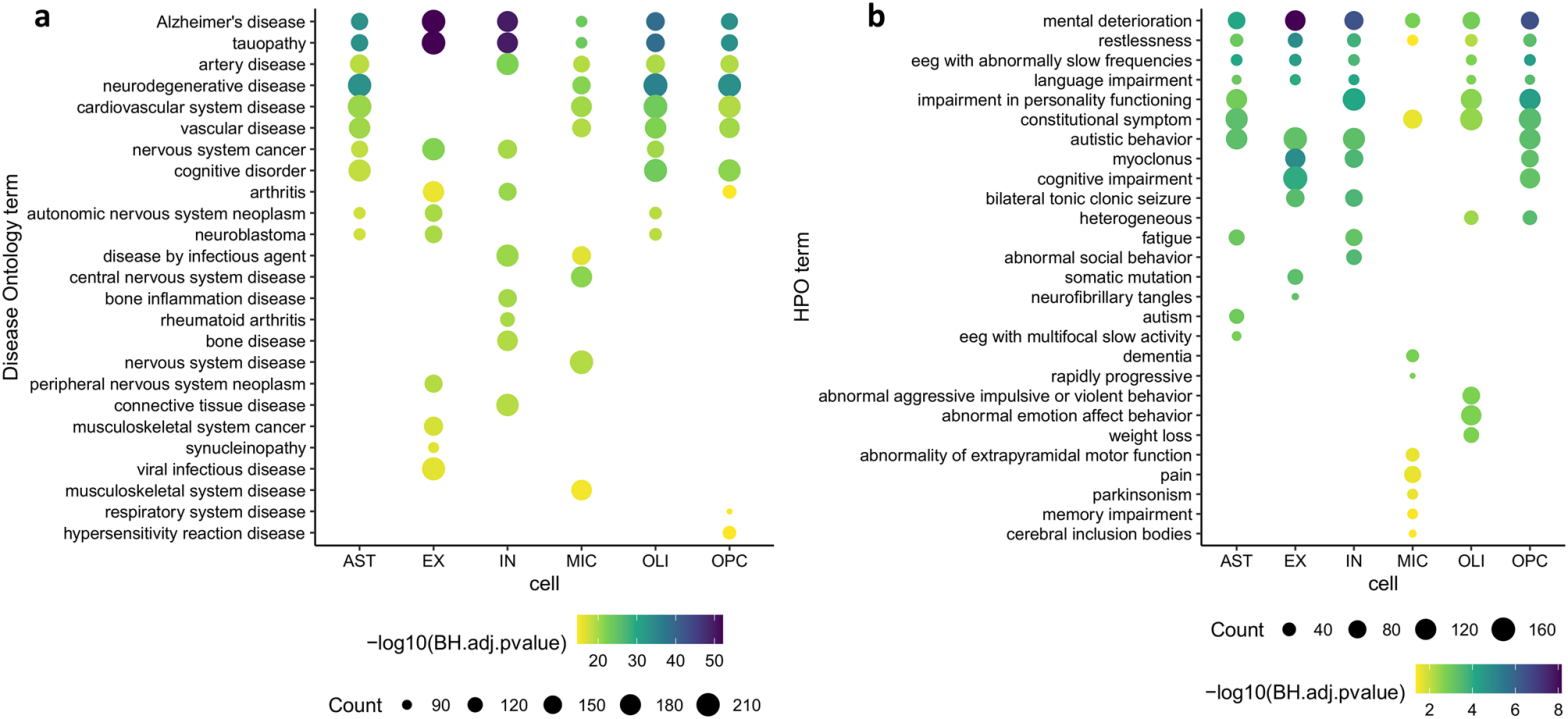
Functional enrichment of the AD genes in Disease Ontology and Human Phenotype Ontology terms. For each cell-specific list of disease genes, the 10 most enriched DO (**a**) and HPO (**b**) terms are shown. On the y-axis the terms are sorted from the top to the bottom according to the number of cells in which they are enriched. The color code indicates the significance of the enrichment, ranging from blue (more significant) to yellow (less significant).

To benchmark the list of AD genes described above, we compared our dataset with two external datasets providing disease gene annotation not used to define our list of AD genes. First, we evaluated the overlap with Alzpedia, a database curated by Alzforum which includes genes and proteins implicated in AD pathophysiology (https://www.alzforum.org/alzpedia). All the AD-related genes included in Alzpedia and expressed in at least one cell type are all present in our list of AD genes. *CD33* and *TDP-43* (*TARDBP*), despite having disease evidence, are not present in the final AD gene list because in the snRNA-seq dataset that we re-analyzed they are below the expression threshold we considered in all cell types (Supplementary Fig. 2). Second, we assessed the enrichment of the AD genes in disease ontology (DO) terms and Human Phenotype Ontology (HPO) gene sets. Overall, the identified DO and HPO enriched terms are consistent with the expected ones. The most enriched DO term is “Alzheimer's disease” (Fig. 2a) while the most enriched HPO term is “mental deterioration” (Fig. 2b). It is worth noting that this analysis was carried out with the aim of assessing the agreement between our list of AD genes and established resources for disease gene annotation, but all the genes in the “AD gene” list were included in the subsequent analyses.

### AD multi-cellular network

We next sought to investigate the AD genes in their cellular context. To build the AD-specific multi-cellular network, we used single-nucleus gene expression data (snRNA-seq) of the human prefrontal cortex from AD patients^21^and publicly available protein–protein and ligand-receptor interaction databases. First, we built a cell-type-specific molecular network for each cell type by combining protein–protein interactions and transcription factor-gene interactions (gene regulatory network—GRN). The GRNs were reconstructed from the AD snRNA-seq data using pySCENIC^29,30^, a tool for reconstructing GRNs from single-cell sequencing data that integrates gene co-expression data with external information on transcription factor binding sites. Each cell-specific GRN was then merged with the protein–protein interaction network keeping only interactions between genes with evidence of expression in the corresponding network. Then, we modularized the obtained network and identified the modules enriched in disease genes (AD modules). Overall, we identified 61 significant AD modules: 9 for the EX network, 4 for the MIC network, 17 for the IN network, 8 for the AST network, 12 for the OLI network, and 11 for the OPC network. The full list of significant AD modules is reported in Supplementary Table 2.

**Figure 3:**
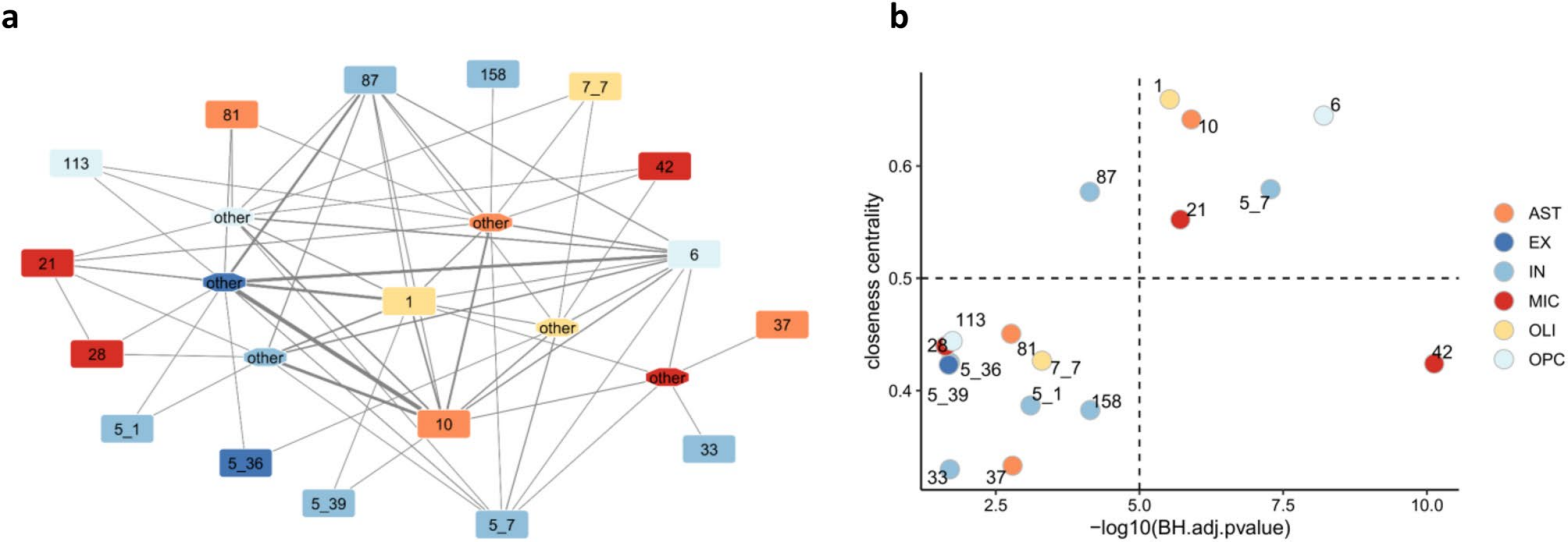
AD multi-cellular network. (**a**) Visualization of the multi-cellular network. Each node corresponds to a cell-specific AD module except for the nodes labeled as “other” which indicate genes not included in any significant AD module but interacting with them thanks to ligand receptor interactions. The edges between the nodes indicate ligand-receptor interactions. The color of the nodes indicates the cell type. (**b**) The significance of the disease gene enrichment for each AD module (x-axis) is plotted versus the network centrality of the corresponding node (y-axis). In the upper right corner are reported the AD modules with the highest significance and a high level of centrality in the network.

To identify the interactions among the AD modules, we analyzed the AD snRNA-seq data using CellPhoneDB^31^, a tool for inferring cell–cell communication networks from single-cell transcriptome data. In total, we identified 57 interacting pairs in which at least one partner is part of a disease module and 41 interacting pairs including at least a disease gene (Supplementary Table 3). A diagram of the workflow followed to build the multicellular AD network is reported in Supplementary Fig. 3. The resulting AD signaling network includes 17 interconnected disease modules, as shown in Fig. 3a. In the network, the nodes labelled with a number indicate AD modules while the nodes labelled as “other”, represent interactions with genes not included in any AD module but present in their corresponding network.

**Figure 4:**
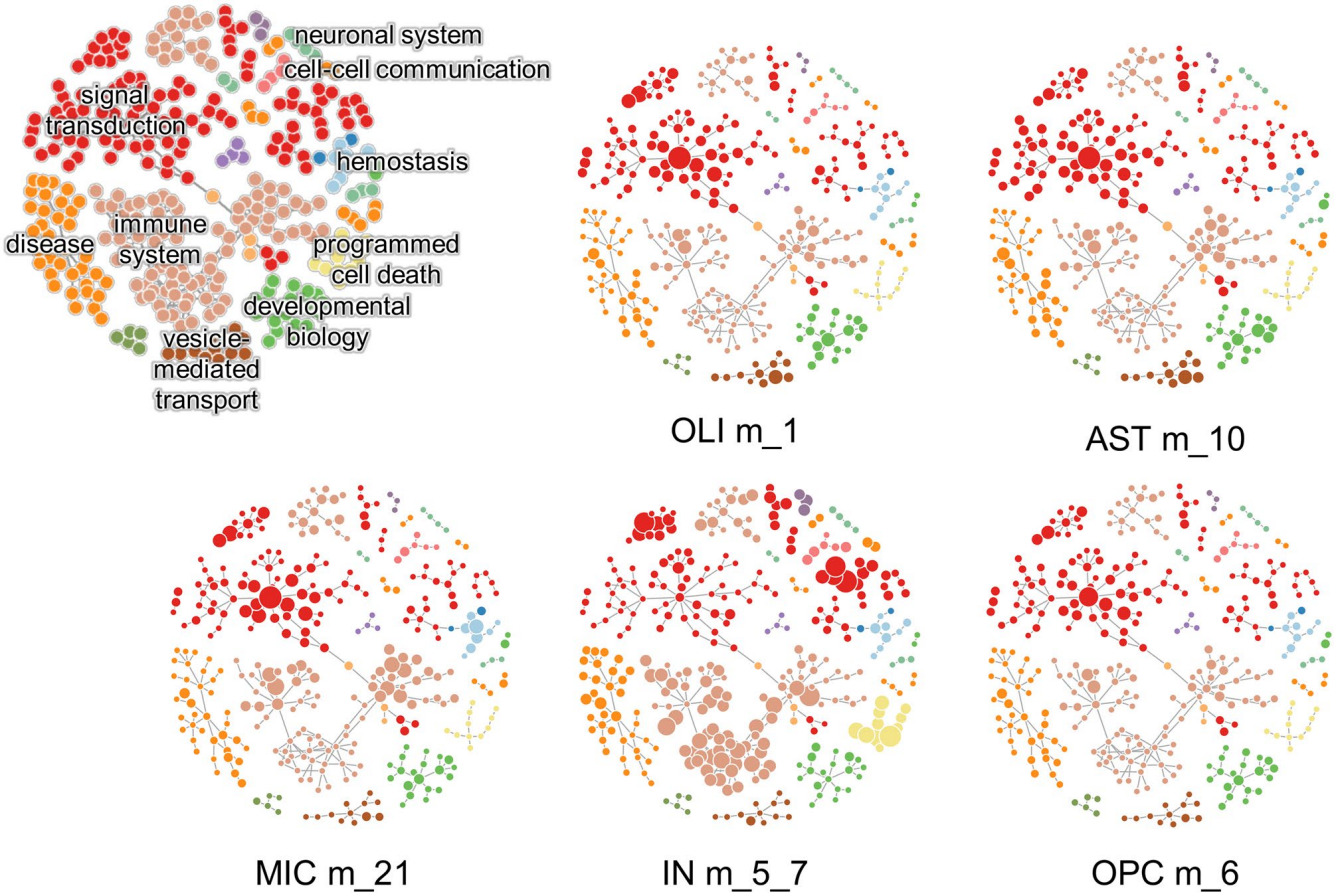
Functional analysis of the core AD modules. Network visualization of the Reactome pathway enrichment analysis results. In each network, the nodes are the pathways and the connections between them reflect the ontology structure of Reactome database with parent–child relationships. Each color corresponds to one ancestor pathway, as shown in the network in the upper left corner where the names of the ancestor pathways are shown. In the other networks the size of the nodes is proportional to the significance of the pathway enrichment test. The complete results are reported in Supplementary Table 3.

Eleven of the interactions present in the multi-cellular network directly connect AD modules. The most interconnected AD module is m_1 from the OLI network. This module is made of 317 genes, 87 of which identified as disease genes and is connected to five AD module genes: OPC m_6, IN m_5_39, AST m_10, IN m_87, MIC m_21 as well as to other non-AD-module genes of all the networks. To assess the influence of each AD module on the entire multi-cellular network, we computed the closeness centrality (CC), a network centrality index that for each node measures the number of steps required to access all the other nodes in the network. By analyzing the relation between the CC and the adjusted p-value of the enrichment in disease genes, we identified five modules with a high CC index and highly enriched in disease genes: OLI m_1, AST m_10, OPC m_6, IN m 5_7, and MIC m_21, hereafter indicated as “core AD modules” (Fig. 3b). As expected by their connectivity, the genes belonging to the core AD modules share many biological functions. According to the Reactome pathway enrichment analysis these modules are mainly involved in signal transduction and in immune system functions (Fig. 4 and Supplementary Table 4).

Since it has been repeatedly reported that therapeutic targets with disease-associated alleles are more likely to be approved^32–35^, we further investigated the disease genes with a supporting genetic evidence belonging to these modules. Overall, we identified 13 genes satisfying this criterion. According to Pharos database^36^, one of them (*PTK2B*) encodes a target with a Tclin target development level (TDL), meaning it exists at least one drug acting by targeting this gene. Out of the other 12 genes, four encode proteins with a Tchem TDL level (they are known to bind small molecules with high potency) and 8 proteins with a Tbio TDL (there is knowledge about their functional role, but they do not have known drug or small molecule activities) (Table 1).

**Table 1:** Genes in the AD core modules with a supporting genetics evidence.

Gene	AD network modules	Disease evidence sources	Pharos tdl	Pharos novelty	Open targets articles
*AP2A2*	AST m_10	GWAS gene, MAGMA pathway (REACTOME INNATE IMMUNE SYSTEM), ARUK	Tbio	0.089	–
*CASS4*	MIC m_21	GWAS gene, ARUK, functional genomics microglia	Tbio	0.01	PMID: 27113998 (drosophila knockdown)
*CD2AP*	MIC m_21, AST m_10, OLI m_1, OPC m_6	GWAS gene, ARUK, functional genomics microglia and oligodendrocytes	Tbio	0.006	PMID: 26358779 (mouse knockout)
*FCER1G*	MIC m_21	GWAS gene, AGORA, functional genomics microglia	Tbio	0.008	–
*HBEGF*	IN m_5_7	GWAS gene, MAGMA pathway (KEGG GNRH SIGNALING PATHWAY)	Tbio	0.001	–
*INPP5D*	MIC m_21	AGORA, functional genomics microglia	Tbio	0.006	–
*MAP3K3*	IN_m_5_7	chromatin neurons	Tchem	0.016	–
*MARK4*	IN_m_5_7	GWAS gene, AGORA, ARUK, functional genomics neurons	Tchem	0.019	–
*PFKFB2*	IN_m_5_7	GWAS gene, functional genomics neurons	Tchem	0.016	–
*PICALM*	AST m_10, OLI m_1	GWAS gene, MAGMA GO (numerous gene sets), ARUK, harmonizome, functional genomics oligodendrocytes	Tbio	0.008	PMID: 26005850 (mouse knockout)
*PLCG2*	MIC m_21, AST m_10, OLI m_1	GWAS gene, AGORA	Tchem	0.003	–
*PTK2B*	MIC m_21, AST m_10, OLI m_1, OPC m_6	GWAS gene, MAGMA pathway (KEGG GNRH SIGNALING PATHWAY), MAGMA GO (GO GLIAL CELL PROLIFERATION), ARUK, functional genomics microglia	Tclin	0.002	PMID: 27113998 (drosophila knockdown)
*SQSTM1*	IN m_5_7	GWAS gene, ARUK, harmonizome	Tbio	0.001	PMID: 32855357 (mouse AD model + *Lactobacillus lactis*)

To complement Pharos information, we also retrieved the AD OpenTargets^37^ scores (Supplementary Fig. 4a). The target with the highest AD overall association score is CD2 Associated Protein, a scaffolding protein that regulates the actin cytoskeleton (encoded by *CD2AP* gene). *PICALM* is instead the gene with the highest text-mining Open Target score. By leveraging the text-mining data used by Open Targets to derive the text-mining score, we identified the genetic targets already evaluated in preclinical studies. The effect of CASS4 and PTK2B on Tau toxicity has been evaluated using fruit fly knockdown models and both proteins emerged as Tau toxicity modulators. PTK2B, specifically, was identified as a strong Tau toxicity suppressor^38^. *CD2AP* and *PICALM* have been studied by many studies and those reported in Open targets describing animal models with highest score were performed using knockout mice^39,40^. Cecarini et al., instead, investigated the neuroprotective effects of SQSTM1 using a *SQSTM1*-engineered *Lactobacillus lactis *orally administered to an AD mouse model^41^. By investigating the brain cell-type specificity of these candidate targets, we observed that the high affinity IgE receptor (gene *FCER1G*) is highly specific for microglia, followed by Inositol Polyphosphate-5-Phosphatase D (gene *INPP5D*) and (Phospholipase C Gamma 2) PLCG2 (Supplementary Fig. 4b), all showing high specificity for microglia.

**Figure 5:**
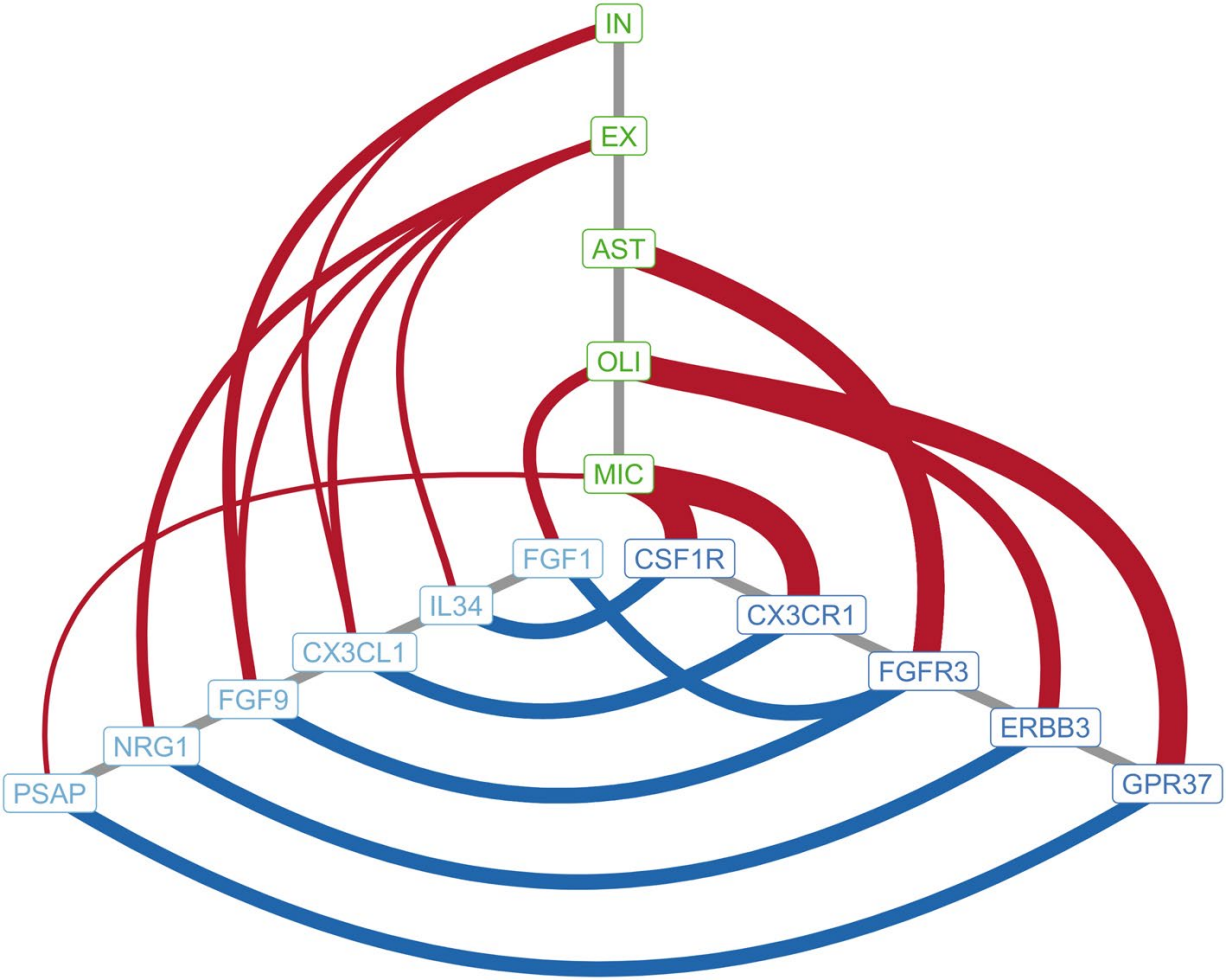
Selected ligand/receptor pairs connecting the modules of the AD multi-cellular network. The genes indicated in light blue encode ligands, those in dark blue encode receptors and the blue line connecting them indicates a significant-ligand receptor interaction inferred from the single cell transcriptomics data. The red lines indicate the cells producing ligands and receptors and its thickness is proportional to the cell specificity of the gene encoding the ligand/receptor.

To further investigate the interaction among the AD modules that are present in the multi-cellular network, we investigated the cell-type specificity of the significant interacting molecules identified by CellPhoneDB (Supplementary Table 3). The most specific ligand-receptor pairs (average specificity > 0.5) with the two partners expressed by different cells (paracrine signaling) included mainly genes of the microglia AD network modules m_21 and m_28 and astrocyte m_10 (Fig. 5). Interestingly, some of these signaling axes have been previously investigated for the identification of new AD treatments in preclinical studies. For example, recent studies investigated the potential of targeting the CSF1R/IL34 axis to reduce the microglial activation and neuroinflammation present in AD^42–44^. The ligand-receptor pair CX3CL1/CX3CR1 in AD was also investigated for its role in AD^45–47^.

### Identification of drug repurposing candidates for AD

To further investigate the therapeutic opportunities for AD, we exploited the network-based drug-disease proximity score we previously defined^17^. This score considers the physical proximity between disease and drug genes in the network as well as their functional similarity. It is computed as the sum of the average closest distance and the gene ontology biological process similarity between the genes in the drug module and genes in the disease module. To identify drug repurposing candidates, we focused on approved drugs with a drug module proximal to one of the core AD modules of the multi-cellular network (Fig. 3a). This choice was guided by the fact that these drugs, thanks to the ligand-receptor connectivity, can influence the entire AD multi-cellular network. To further filter the results, the drugs with a target that has a high level of specificity (Human Protein Atlas tissue/group enriched/enhanced) for a tissue other than those relevant for the disease have been excluded. In total, we identified 54 candidate drugs targeting 37 proteins (Table 2). For each of them we retrieved the current disease indications from ChEMBL database. This analysis indicated that six of them, namely Acitretin, Bromocriptine, Caffeine, Dasatinib, Doconexent, and Nilotinib, have already been investigated in clinical trials of AD. Acitretin was tested in a small clinical trial for its ability to enhance neuronal a-secretase ADAM10 activity and thus APP levels in CSF (NCT01078168^48^). Bromocriptine was recently tested in phase I/IIa study of familial AD (NCT04413344^49^) and an ongoing clinical trial (NCT04570085) is currently evaluating the caffeine efficacy on cognitive decline in Alzheimer's disease dementia. Dasatinib is under investigation for its ability, in combination with quercetin, to selectively remove senescent cells from the Aβ plaque (NCT04063124^50^). Doconexent is an omega 3 fatty acid used as nutritional supplement that has been tested in a randomized clinical trial for its ability to slow the rate of cognitive and functional decline in the general population of AD patients (NCT00440050^51^) and in APOE4 allele carriers (NCT03613844^52^). Finally, a phase 2 clinical trial testing the impact of Nilotinib in mild AD is also reported in clinicaltrials.gov database. A more detailed description of AD clinical and preclinical studies of the identified drugs is reported in Supplementary Note 1 and the Open Targets association score with AD is reported in Supplementary Table 5.

**Table 2:** Drug repurposing results.

Drug target	Drugs	Modules
ABL1	Bosutinib, Dasatinib, Nilotinib, Ponatinib	AST m_10, MIC m_21, OLI m_1, OPC m_6
ADORA1	Caffeine	OLI m_1
ADRB2	Isoprenaline, Salbutamol	MIC m_21
ALK	Crizotinib	IN m_5_7, OLI m_1, OPC m_6
BCR	Bosutinib, Imatinib, Ponatinib	AST m_10, IN m_5_7, OLI m_1, OPC m_6
DHFR	Methotrexate, Proguanil	IN m_5_7
DRD3	Bromocriptine	IN m_5_7
ERBB2	Afatinib, Lapatinib	AST m_10
FGF2	Sirolimus	AST m_10, OLI m_1
FGFR1	Nintedanib, Sorafenib	IN m_5_7, MIC m_21, OLI m_1, OPC m_6
FGFR2	Nintedanib	OLI m_1
FLT3	Midostaurin	OLI m_1
GABRA1	Acamprosate, Flumazenil, Nitrazepam, Pentobarbital, Primidone, Propofol	IN m_5_7
HDAC1	Belinostat	IN m_5_7, OLI m_1
HTR2A	Asenapine, Dosulepin	IN m_5_7
IKBKB	Auranofin	AST m_10, IN m_5_7
JAK1	Ruxolitinib, Tofacitinib	AST m_10, MIC m_21, OLI m_1, OPC m_6
JAK2	Ruxolitinib, Tofacitinib	AST m_10, IN m_5_7, MIC m_21, OLI m_1, OPC m_6
JUN	Adapalene	AST m_10
KDR	Midostaurin, Pazopanib	OLI m_1
MAP2K1	Selumetinib	AST m_10, MIC m_21
MAP2K2	Selumetinib, Trametinib	AST m_10, OLI m_1, OPC m_6
MAPT	Paclitaxel	IN m_5_7
MTOR	Everolimus	IN m_5_7
NR3C1	Budesonide, Clocortolone, Fluorometholone, Hydrocortisone	AST m_10, IN m_5_7, MIC m_21
NR3C2	Eplerenone, Spironolactone	AST m_10, IN m_5_7
OPRD1	Loperamide, Naloxone	IN m_5_7
OPRK1	Loperamide	IN m_5_7
PDE4A	Dipyridamole, Dyphylline, Theophylline	IN m_5_7, OPC m_6
PDE5A	Dipyridamole, Tadalafil, Theophylline	AST m_10, IN m_5_7, OPC m_6
PDGFRB	Pazopanib, Sorafenib, Sunitinib	AST m_10, IN m_5_7, OPC m_6
PPARA	Bezafibrate, Doconexent	IN m_5_7, OLI m_1, OPC m_6
RAF1	Dabrafenib	AST m_10
RARA	Acitretin	IN m_5_7
RRM2B	Cladribine	IN m_5_7
RXRG	Adapalene	IN m_5_7
SRC	Dasatinib	AST m_10, IN m_5_7

**Figure 6:**
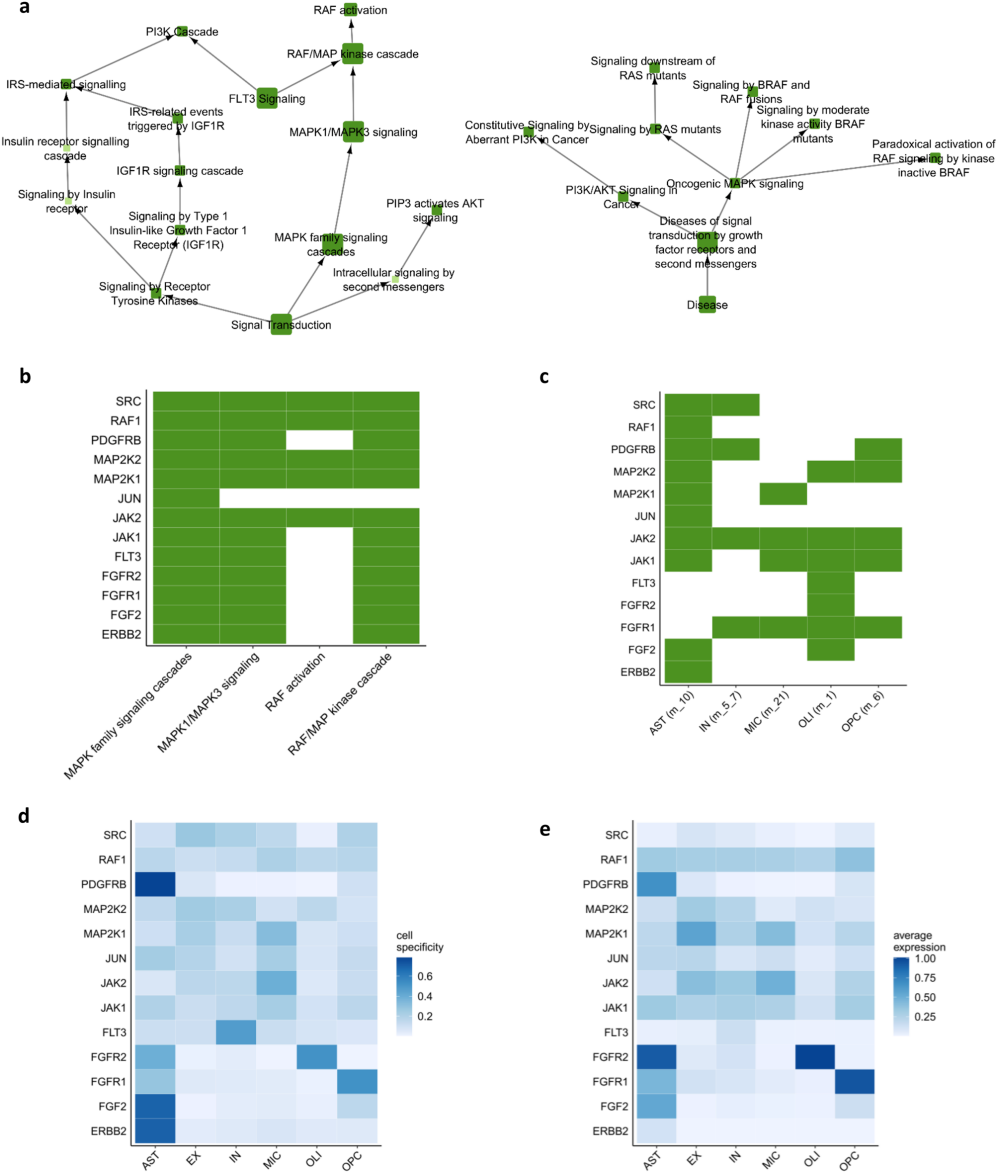
Characterization of the drug targets identified by the drug repurposing pipeline. (**a**) Results of Reactome pathway enrichment analysis. Each node corresponds to one pathway and its size is proportional to the significance of the enrichment. The connections between pathways reflect the ontology structure of Reactome database with parent–child relationships. The small light green circles correspond to pathways not significantly enriched but needed to keep the network connected. The two ancestor pathways of the significantly enriched pathways are Signal Transduction (on the left) and Disease (on the right). (**b**) Detailed visualization of the genes belonging to MAPK-related Reactome pathways. (**c**) Chart showing the cell networks in which the targets belonging to the MAPK pathways were identified as significantly proximal to AD modules. The corresponding AD module is indicated in parenthesis next to the cell name. (**d**) Cell specificity of the targets belonging to the MAPK pathways. (**e**) Average expression in each cell of the targets belonging to the MAPK pathways.

Pathway analysis of the 37 drug targets showed that mitogen-activated protein kinase (MAPK) and, to a lesser extent, Insulin Like Growth Factor 1 Receptor (IGF1R) signaling were the most enriched (Fig. 6a and Supplementary Table 6). Specifically, the identified targets mapped to the signaling cascade leading to the activation of RAS, RAF, and the MAPK kinase proteins (Fig. 6b).

Since the identified targets are present in more than one cell networks, we evaluated the target cell specificity in AD cells using the score provided by the Expression Weighted Cell type Enrichment (EWCE) package. Even if some of the targets and their corresponding drug module were identified as significantly associated to more than one disease module, the cell specificity analysis suggests a preferential expression of the gene in one cell type. For example, PDGFRG was identified as significantly associated with disease modules of AST, IN, and OPC, however the gene resulted AST-specific in AD cells. Similarly, FGF2 and ERBB2 are AST-specific, despite being expressed by other cell types (Fig. 6d,e).

## Discussion

To date, only a few drugs for AD, mainly targeting the disease symptoms, are available, and thus identifying new treatment options is crucial. In recent years, numerous clinical trials of disease-modifying therapeutics for AD have been conducted but they mostly led to negative results^9,10^. In this contest, drug repurposing can be exploited for the identification of new AD drug candidates.

Here we described a systems biology workflow based on single-cell gene expression to identify drug repurposing candidates for AD. First, we identified the AD genes using several evidence sources which include genetics, gene expression, and literature. This allowed us to cover the different approaches that can lead to the identification of the genes involved in AD, such as data-driven high-throughput studies (e.g., GWAS and differential gene expression analysis of single-cell RNA-seq data) as well as hypothesis-driven functional studies reported in the literature.

Cell-specific networks were built to contextualize the identified genes in their biological pathways and identify drug repurposing candidates. The network approach we adopted allowed us to move the focus from the individual genes to the disease modules and point out molecular mechanisms involving more than one AD-associated gene, reflecting the propensity of disease genes to cluster in network modules^53,54^. The molecular networks were built by combining cell-specific TF-gene interactions derived from the GRN inferred from the transcriptomics data with protein–protein interactions retrieved from the literature. This allowed us to obtain a comprehensive description of the functional context in which the gene products are involved. Moreover, by adopting a cell-specific approach, the identified pathways are linked to a specific brain cell, and, thanks to the analysis of the ligand-receptor interactions, cell–cell communication can also be investigated. The AD multi-cellular network we built is an interconnected set of disease modules that communicate through ligand-receptor interactions that we predicted from the gene expression data. Thus, by perturbing one of the disease modules, especially the most central ones, it is possible to affect other cells that are involved in the disease. This is of particular interest in pathological conditions affecting multiple cell types, such as AD.

Our repurposing results suggest that perturbing the MAPK pathway may be an effective strategy for the treatment of AD. Recent studies underlined the importance of this pathway in the disease pathophysiology, in particular concerning neuroinflammation, and highlighted the potential benefit of MAPK inhibitor for its treatment^55–57^. Among the identified drugs targeting the MAPK pathway and other related pathways, afatinib and lapatinib are cancer drugs that inhibit tyrosine kinase receptors belonging to the EGFR family, reported by the Reactome pathway database as being involved in MAPK upstream signaling. A potential indication for their efficacy in AD derives from evidence for a putative role of the EGFR pathway in suppressing autophagy and the demonstration that its inhibition decreased amyloid-β secretion in vitro and in vivo and improved cognitive functions in AD models^58,59^. In line with these findings, lapatinib reversed memory deficits in a mouse model of cognitive impairment^60^ whereas afatinib efficacy in contrasting neuroinflammation suggested a potential efficacy in neurodegenerative diseases^61^. Nintedanib and sorafenib are antitumoral agents acting on multiple tyrosine kinase targets which exert their action also by modulating tumor-mediated angiogenesis. In our study, they were identified as AD repurposing candidates thanks to the proximity of FGFR1 and FGFR2 drug modules to disease modules of oligodendrocytes, oligodendrocyte progenitor cells, microglia, and inhibitory neurons. Indications supporting repurposing for AD therapy derive from demonstrated efficacy in diminishing neuroinflammatory responses and restoring cognitive abilities in AD mice models^62,63^. Since Raf inhibition has been suggested as a relevant mechanistic target for these responses^64^, the Raf inhibitor dabrafenib may also represent a promising drug. Similarly, JAKs inhibitors ruxolitinib and tofacitinib potential efficacy is related to efficacy in dampening excessive inflammatory responses and have therefore already been suggested as objects of repurposing efforts^65,66^. MEK inhibitors selumetinib and trametinib could also possibly act on AD-associated neuroinflammation. Since TREM2 loss of function is one of the strongest known genetic AD risk factors, the discovery that MEK inhibition was able to raise TREM2 cell surface expression and function indicates opportunities for therapeutic intervention with these agents^67^. Sunitinib, which inhibits several tyrosine kinase receptors, has been previously associated to AD therapy with different mechanisms. Indeed, Lee et al*.* identified sunitinib in screenings aimed at identifying molecules able to dissociate Aβ oligomers and plaques to monomers in 5XFAD transgenic mice^68^. However, sunitinib has also been reported to act as an anticholinesterase inhibitor and to attenuate scopolamine-induced impairments of learning and memory in mice similarly to donepezil^69^. Moreover, reversal of AD-associated vascular activation was suggested as a mechanism supporting sunitinib-induced improvement in cognitive functions observed in AD mice models^70^. It is noteworthy that the list of genes from the AD core modules with supporting genetic evidence (Table 1) includes a gene of the MAPK pathway, *MAP3K3.* This gene encodes MEKK3, a kinase acting upstream of ERK5 described as a positive regulator of mitophagy^71^. Interestingly, the dysfunction of the autophagy-lysosomal system and in particular the mitophagy impairment has been observed in AD patients and AD animal models^72^, and the dysregulation of the MAPK signaling pathway could be involved in this process.

Of note, among the repurposing candidates we identified also drugs targeting pathways outside the MAPK and IGF1R signaling. For example, the protein with the highest Open Targets AD overall association score identified by the repurposing pipeline is the serotonin receptor HTR2A, target of Asenapine and Dosulepin. Serotonin alterations have been implicated in AD development, however the potential therapeutic effect of drugs targeting this pathway needs to be further investigated^73^. In addition to the repurposing candidates, our approach allowed us to identify genes linked to AD based on different lines of evidence and map them to their molecular and cellular context. In particular, the genes reported in Table 1, identified as AD genes with genetic evidence in the AD multicellular network, are those we consider more promising, and they could be further investigated using in vitro and in vivo models.

In conclusion, the approach described in this study allowed us to gain cell-specific insights into AD molecular mechanisms that were translated into drug repurposing hypotheses that could be further evaluated. A limitation of this investigation is the relatively small cohort of AD subjects in the single-cell RNA-seq that may not consider the great heterogeneity among subjects at different disease stages. In the future, the inclusion of new AD datasets that will be available in the public domain could broaden the results presented here and lead to the identification of additional drug targets and repurposing candidates. Like other computational approaches, the results of this study would need to be tested in experimental settings to evaluate their relevance. In the current version of the pipeline, the drugs with a significant repurposing score indicate both possible disease indication or drug side effect and we identified the most promising candidates based on literature evidence. Future improvements of the pipeline could focus on the inclusion of information about the directionality in the repurposing score. Moreover, the framework we developed can be easily adapted to other diseases for which is possible to define a list of disease-associated genes.

## Methods

### Analysis of the snRNA-seq data of prefrontal cortex

The brain snRNA-seq dataset analyzed in this study was obtained from Mathys et al.^21^ (downloaded on 13/02/2020 from https://www.synapse.org/#!Synapse:syn18485175). The use of this dataset for the project has been approved by Synapse on 06/12/2019. This dataset is provided by the Rush Alzheimer’s Disease Center, Rush University Medical Center, Chicago. Data collection was supported through funding by NIA grants P30AG10161, R01AG15819, R01AG17917, R01AG30146, R01AG36836, U01AG32984, U01AG46152, the Illinois Department of Public Health, and the Translational Genomics Research Institute. It comprises snRNA-seq data from post-mortem human brain samples from 48 participants in the Religious Order Study (ROS) or the Rush Memory and Aging Project (MAP), two longitudinal cohort studies of ageing and dementia. The Brodmann area 10 prefrontal cortex tissue from 24 individuals with high levels of β-amyloid and other pathological hallmarks of AD (‘AD-pathology’), and 24 individuals with no or very low β-amyloid burden or other pathologies (‘no-pathology’) were selected for snRNA-seq. We based our analyses on the already pre-processed, filtered data present in the Synapse repository. 17,926 genes profiled in 75,060 nuclei. The dataset includes eight main cell types: excitatory neurons, inhibitory neurons, astrocytes, oligodendrocytes, microglia, oligodendrocyte progenitor cells, pericytes and endocytes. Pericytes and endocytes data were excluded from the analysis because of the lower cell counts, in agreement with the original publication^21^. This dataset was analyzed using the Seurat R package (v. 4.0). The cell type specificity of the genes was assessed using the EWCE R package^74^. To identify the genes to include in the cell-specific networks, an additional filtering was performed. Cells from AD subjects and controls were analyzed separately and we considered a gene as expressed if at least two cells for each cell type had more than one count. The ligand-receptor analysis was performed using the python package CellPhoneDB^31^.

### Identification of AD genes from GWAS summary statistics (genetic evidence)

The AD GWAS summary statistics included in this study were published in 2019 by Kunkle et al.^23^ and Jansen et al.^22^. The datasets were downloaded from https://ctg.cncr.nl/software/summary_statistics and https://www.niagads.org/datasets/ng00075 on 4^th^ February 2020. To aggregate the SNP association signals into gene and pathway association signals, we run MAGMA v1.07b, a tool for gene and gene-set analysis of GWAS results^24^. The file with the chromosome position of the SNPs (GENELOC_FILE) (hg 19) and the linkage disequilibrium file (1,000 Genomes European panel) were downloaded from MAGMA website on 10^th^ February 2020. The gene-set files (curated gene sets and gene ontology GO biological processes) were downloaded from MSigDB (https://www.gsea-msigdb.org/gsea/msigdb/) on 10th February 2020. We corrected the p-values of the genes and the pathways using the Benjamini & Hochberg (BH) multiple-test correction method. To identify significant genes and pathways, we set a threshold of 0.05 for the BH adjusted p-value. The GWAS summary statistics were also translated into gene-level statistics by assigning risk variants to their putative genes based on tissue-specific eQTL information using E-MAGMA^25^. We run E-MAGMA using the brain cortex GTEx (v7) genes expression data, downloaded from E-MAGMA repository (https://github.com/eskederks/eMAGMA-tutorial). We corrected the p-value for multiple testing using the BH correction and selected as significant the genes with a corrected p-value below the threshold of 0.05. We also retrieve AD genes with genetics evidence from a published functional genomics study^26^. In this work, AD genes were identified according to whether they had active promoters/enhancers that overlapped with AD risk variants from. The dataset is cell-specific and it included AD genes related to neurons (both excitatory and inhibitory), microglia and oligodendrocytes^26^.

### Identification of AD genes from differential expression analysis of snRNA-seq data (gene expression evidence)

The cell-specific lists of differentially expressed genes (DEGs) were obtained from the supplementary materials of Mathys et al.^21^. In agreement with the original publication, we considered DEGs genes that passed both a cell-level analysis test (performed using the Wilcoxon ranksum test and FDR multiple-testing correction) and a Poisson mixed model test model (accounting for the individual of origin for nuclei and for unwanted sources of variability).

### Identification of AD genes from the literature (literature evidence)

We obtained AD genes from (1) Harmonizome (https://amp.pharm.mssm.edu/Harmonizome), (2) KEGG AD pathway (hsa05010), (3) Agorà, a list of over 500 candidate drug targets for AD that were nominated by different groups of AD researchers (https://agora.ampadportal.org/), (4) AD Gene Ontology terms from Alzheimer's Disease Gene Ontology Annotation Initiative of Alzheimer's Research UK (https://www.ebi.ac.uk/GOA/ARUK).

### Scoring of the disease genes

A scoring system was used to rank the AD genes based on the number of different sources of evidence supporting the association. A 0/1 score was assigned to each gene based on the absence/presence of the gene in each main source of evidence (genetics, gene expression, literature). Genes having only genetic evidence derived from MAGMA annotation (genomic proximity) and no functional evidence were not considered as disease genes because, due to linkage disequilibrium, the genes in a genomic risk locus corresponding to GWAS hit are not all necessarily involved in the disease. In addition, we computed a diversification score by assigning a higher impact to an AD gene if we found different sources of evidence supporting the association. The diversification score was computed as 1—Gini index using the Gini function from the DescTools R package. The final disease score was obtained by summing the subcomponents.

### Evaluation of the AD gene dataset using other annotations

The relevance of the genes we included in the dataset of AD genes was assessed using three established resources providing gene annotations: (1) AlzPedia, an AD-specific resource containing genes and proteins implicated in the pathophysiology of AD (https://www.alzforum.org/alzpedia), (2) Human Phenotype Ontology (HPO), an ontology of human disease phenotypes^75^, (3) Disease Ontology (DO), an ontology of human disease terms^76^. The enrichment of the AD genes in HPO and DO terms was evaluated using the R package clusterProfiler (v. 3.16.1). While for DO we used the version present in clusterProfiler, the HPO ontology (v. 7.4) was downloaded from the Molecular Signatures Database (MSigDB) (https://www.gsea-msigdb.org/gsea/msigdb/) on 12^th^ May 2021.

### Construction of the AD multi-cellular network

For each cell type present in the snRNA-seq dataset, we built a molecular network including protein–protein and transcription factor (TF)-gene interactions. We retrieved the protein–protein interactions (PPI) from the following resources: HENA^77^ (https://figshare.com/search?q=hena) and Hippie^78^ (http://cbdm-01.zdv.uni-mainz.de/~mschaefer/hippie/). From HENA we downloaded four datasets: PPI of brain ageing (PBA), PPI from IntAct in humans, Alzheimer’s disease PPI from IntAct and synaptic PPI from IntAct (downloaded on 20th February 2020). We filtered each dataset to keep only the most specific interactions: PBA interactions with PBS score < 1, IntAct interactions with MI score between > 0.45. For excitatory and inhibitory neurons, we merged the four sources while for the other cells we did not include the synaptic PPI networks. From Hippie (data downloaded on 27th February 2020), we selected only the high-confidence PPIs (score > = 0.73). To generate cell-specific networks, the resulting network was filtered to keep only the genes which are expressed in the AD dataset (see methods section Analysis of the single-nucleus RNA-seq data of prefrontal cortex for the filtering criteria) or in the control dataset if the gene is differentially expressed genes and downregulated in AD cells (i.e. DEGs that are expressed in controls but not in AD-derived cells). Following this approach, we obtained six cell-specific PPI networks that were merged with the TF-gene interactions (gene regulatory network GRN) inferred from the snRNA-seq data. The GRN was inferred using the pySCENIC pipeline v. 0.9.18^30^, run using the default parameters. To perform the analysis the following databases were used: cisTarget database hg38__refseq-r80__500bp_up_and_100bp_down_tss.mc9nr.feather, transcription factor motif annotation database: motifs-v9-nr.hgnc-m0.001-o0.0.tbl, list of human TF: allTFs_hg38.txt. The files were downloaded on 2nd March 2020.

Network modules were detected using the walktrap algorithm, as implemented in the R package igraph. To avoid oversized modules, the algorithm was run iteratively on modules with more than 500 genes. The enrichment in AD genes was tested using one-sided Fisher’s exact test, followed BH correction for multiple test (corrected *p*-value < 0.05).

The multi-cellular AD network was built by connecting the AD modules based on the number of shared LR pairs. Closeness centrality of the nodes in the multi-cellular AD network was computed using the closeness function from igraph R package.

### Functional enrichment analysis of the genes in the disease modules and the candidate drug targets

We performed the Reacome enrichment analysis using the function enrichPathway from R package ReactomePA. For the modules analysis, all the genes in the corresponding network were used as the universe. For the analysis of the drug targets, instead, all the genes encoding drug targets that we tested in the repurposing pipeline and for which we could build a drug module were used as universe. Pathways with a BH-adjusted *p*-value < 0.05 were considered significantly enriched.

### Annotation of the disease genes with supporting genetic evidence

Pharos database^36^ was queried via the GraphQL API (accessed on 3rd June 2021). Open Targets database^37^ was accessed via the web interface (https://www.opentargets.org/) on 4th May 2021.

### Retrieval of drug information

Information about drugs, their stage of development and their targets was obtained from DrugBank (database downloaded on 9th February 2021). Data was parsed using the dbparser R package. Drugs were retained if they were annotated to have a polypeptide target with a known action and if they had an approved status for humans. The expression profiles of the drugs were obtained from the Library of Integrated Network-Based Cellular Signatures (LINCS). We accessed the data using the RESTful API (https://clue.io/) on 16^th^ February 2021 and for each drug the 100 most up- and down- regulated genes were retrieved relying on high quality signatures (is_gold = 1). If for a certain drug more than one signature was available, we selected the one with the highest signature strength (distil_ss parameter).

### Identification of drug repurposing candidates

To identify drug repurposing candidates, we followed the approach described in in Misselbeck et al.^17^. Briefly, for each drug target-drug pair we built a drug module which includes the drug target, drug modulated genes and connecting genes. Drug modules with less than ten genes were not considered for the identification of repurposing candidates. The repurposing candidates were identified by computing the proximity score between disease and drug modules.

Tissue specificity was evaluated using the gene expression information from Human Protein Atlas (accessed on 15th April 2021). Drug repurposing candidates significantly associated with a disease module of EX, IN, OLI, OPC, or AST network and having a target that is specific (Tissue enriched, Tissue enhanced, Group enriched, Group enhanced) of non-brain tissues were excluded. For MIC network, drug targets specific for non-brain tissues and non-immune-related tissues (lymphoid tissue, blood, bone marrow, gallbladder), were excluded. Cell specificity was calculated using the generate_celltype_data function of the EWCE package using as input the snRNA-seq data from cells of AD subjects. ChEMBL annotation of the disease indications was retrieved using ChEMBL API service. The ChEMBL ids of the drugs were identified from UniChem database using the DrugBank id for the search. The analysis was performed on 12^th^ May 2022.

## Supplementary Information

Supplementary Information 1.

Supplementary Information 2.

Supplementary Information 3.

Supplementary Information 4.

Supplementary Information 5.

Supplementary Information 6.

Supplementary Information 7.

Supplementary Information 8.

## Data Availability

All the data used in this study are publicly available. The brain snRNA-seq dataset analyzed in this study is available from https://www.synapse.org/#!Synapse:syn18485175. The GWAS summary statistics are available from https://ctg.cncr.nl/software/summary_statistics and https://www.niagads.org/datasets/ng00075.

## Code availability

The code to build the AD multicellular network is available from https://www.cosbi.eu/fx/2093489320/. The code to run the repurposing pipeline is available from our previous publication (https://doi.org/10.1038/s41467-019-13208-z).
