# Supplementary material for: Single-cell-led drug repurposing for Alzheimer’s disease
Source: Sci Rep. 2023 Jan 5;13:222. doi: 10.1038/s41598-023-27420-x (PMC9816180; doi:10.1038/s41598-023-27420-x)
Supplement: Supplementary file 7 — Supplementary Information 7. [file 41598_2023_27420_MOESM7_ESM.pdf]

Supplementary Information

Single-cell-led drug repurposing for Alzheimer's disease

Silvia Parolo[1], Federica Mariotti[1], Pranami Bora[1], Lucia Carboni[2], Enrico Domenici[1,3]

[1] Fondazione The Microsoft Research-University of Trento Centre for Computational and Systems Biology (COSBI), 38068 Rovereto, Italy

[2] Department of Pharmacy and Biotechnology, Alma Mater Studiorum University of Bologna, 40126 Bologna, Italy.

[3] Department of Cellular, Computational and Integrative Biology (CIBIO), University of Trento, 38123 Trento, Italy

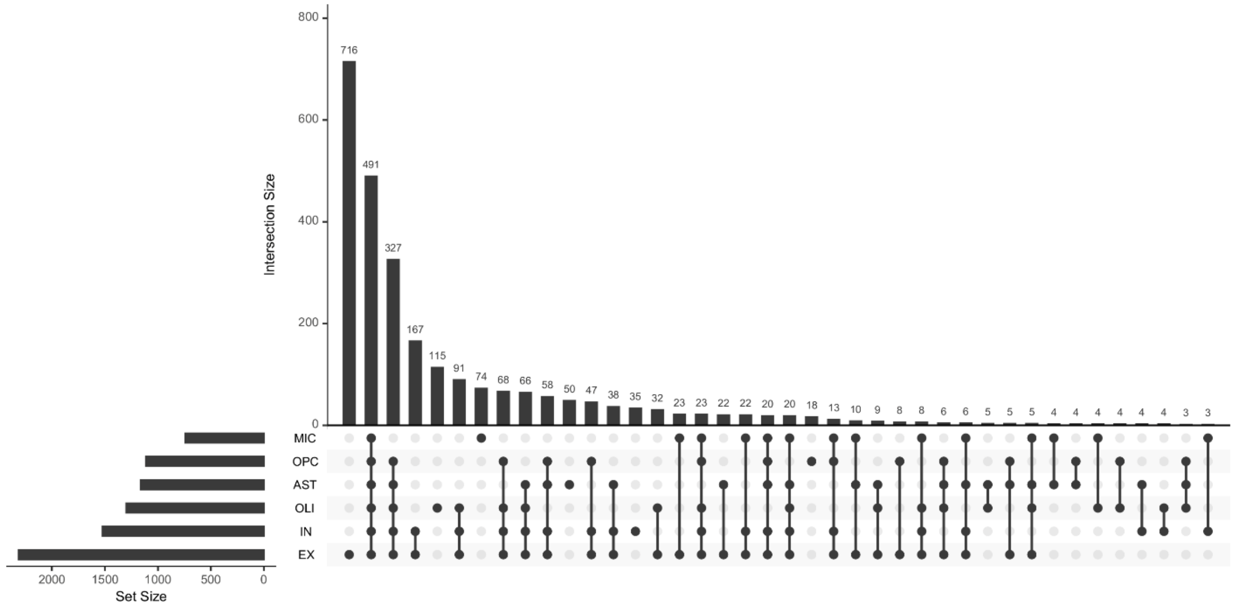

Supplementary Figure 1. Upset plot showing the distribution of disease genes across the six cell types. The horizontal bars on the left show the total number of AD genes for each cell type

while the vertical bars show the number of shared genes for the cells indicated by the points in the matrix.

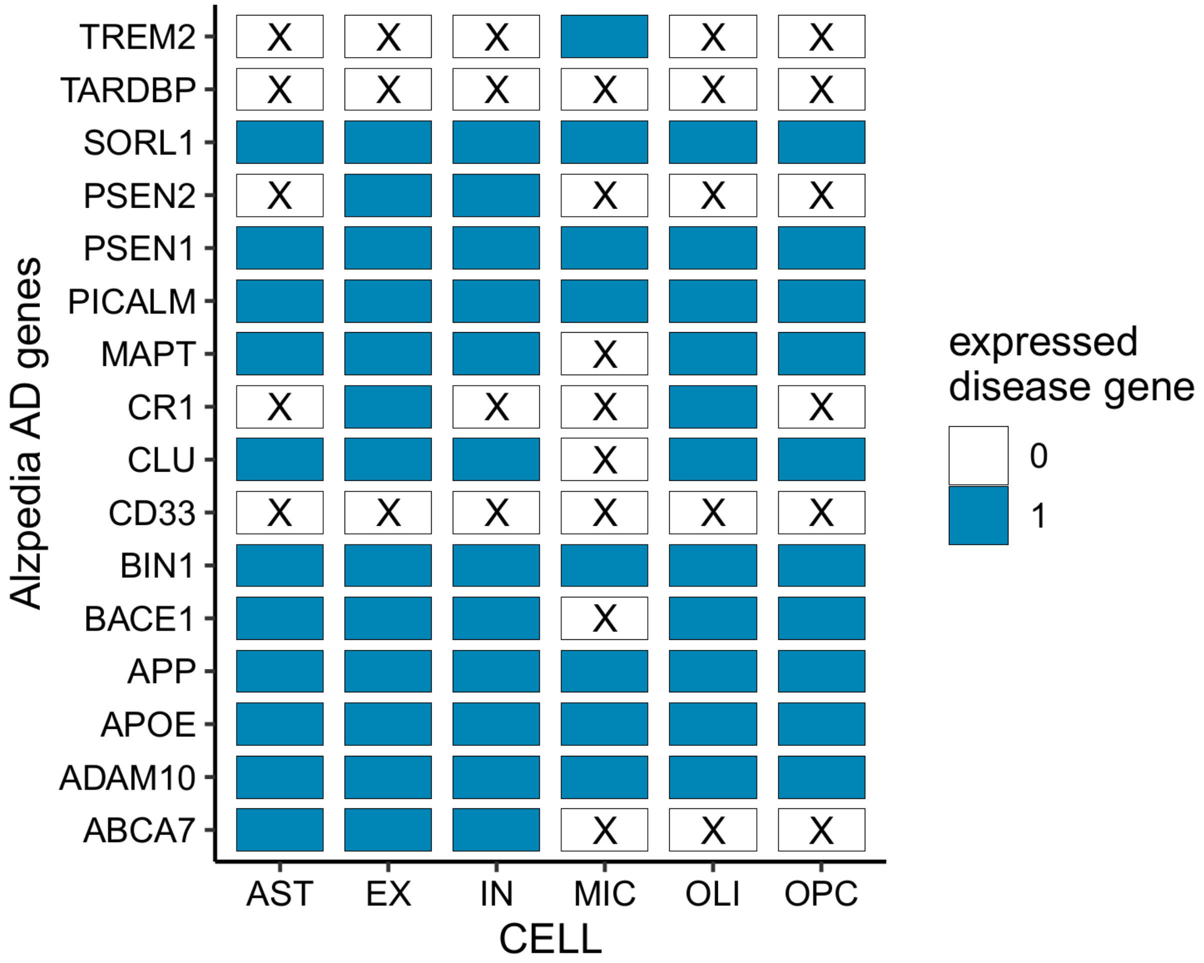

Supplementary Figure 2. AD genes from Alzpedia that are present in our list of AD genes. The X within the boxes indicates a gene not included in the corresponding list of disease genes. We can observe that all X correspond to white boxes, indicating that according to our analysis the gene is not expressed in the corresponding cell type.

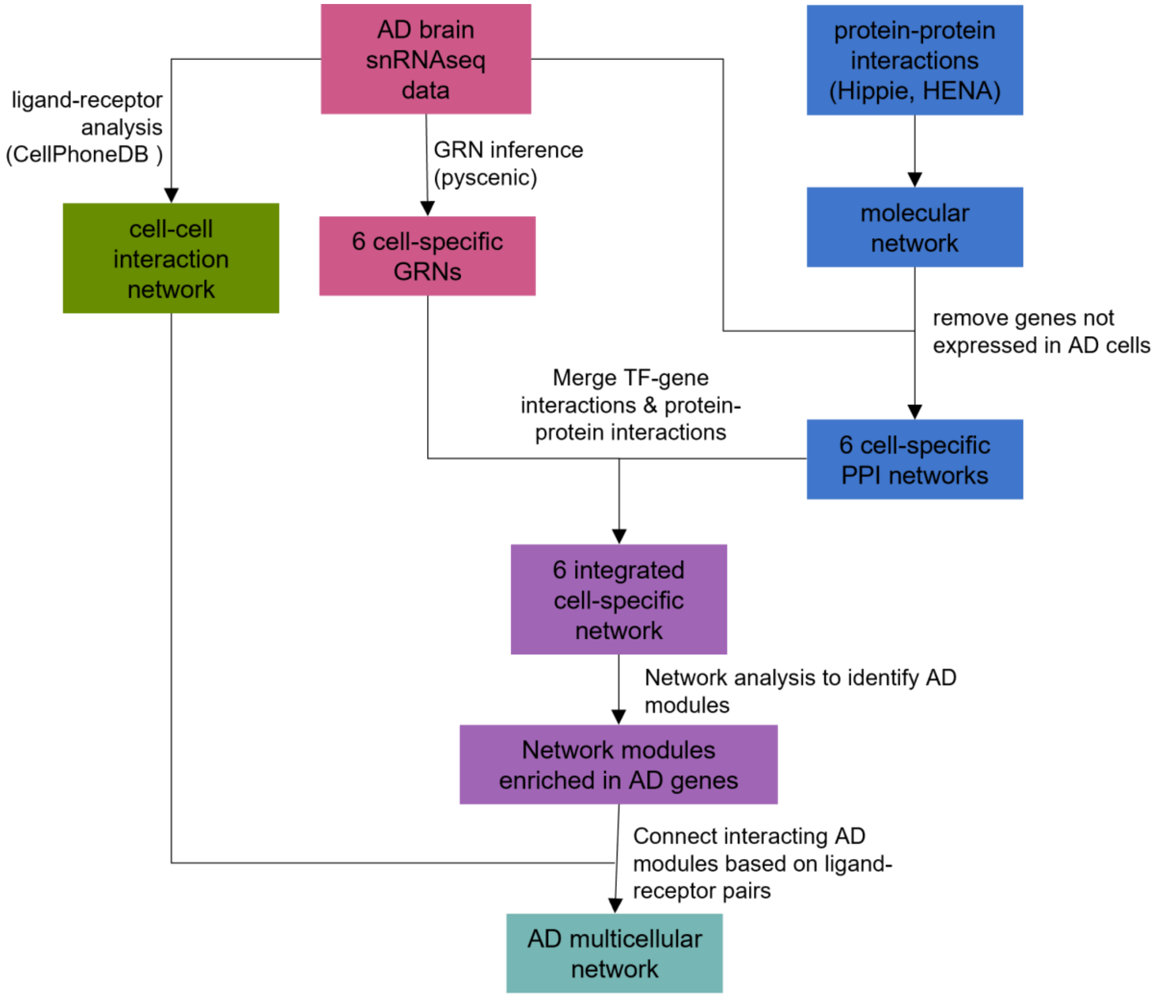

Supplementary Figure 3. Diagram showing the approach followed to build the AD multicellular network. Single nucleus RNA-seq data of AD brain has been used to infer the cell specific gene regulatory networks (pySCENIC tool) and to infer a cell-cell communication network among the six cell types (CellPhoneDB tool). Publicly available protein-protein interactions have been merged with the TF-gene interactions from the gene regulatory networks and 6 cell-specific integrated networks have been obtained. Using the random walk algorithm, we defined network modules, and we tested their enrichment in AD genes. The enriched modules (AD modules) have been connected based on the ligand-receptor interactions inferred from the snRNAseq data thus obtaining the AD multicellular network.

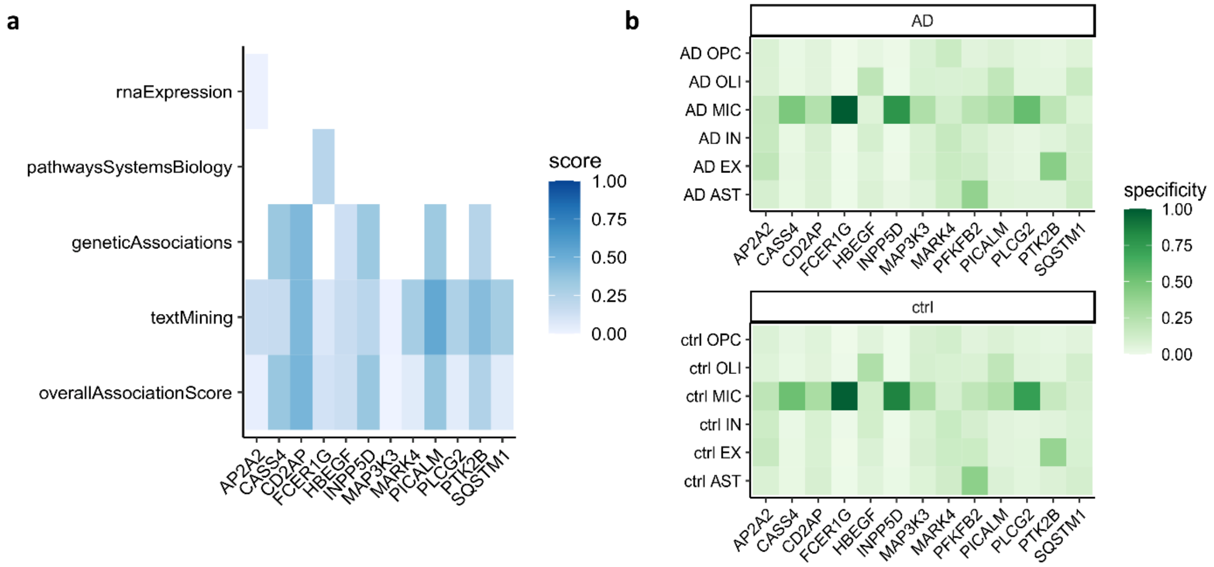

Supplementary Figure 4. Open Targets annotation and cell specificity of AD genes with genetic evidence. a For each AD gene with a supporting genetic evidence, the AD Open Targets association scores are shown. **b** The cell specificity in AD and control cells computed from the single cell transcriptomics data is shown.

Supplementary Note 1

In the following, we report literature information related to the investigation of the most promising candidate repurposing drugs (Table 2 of the main text) in preclinical and clinical studies of AD.

Imatinib, dasatinib, nilotinib, bosutinib, and ponatinib

Imatinib belongs to the first generation of BCR-ABL kinase inhibitors, followed by second generation inhibitors dasatinib, nilotinib, and bosutinib, and by third generation inhibitor ponatinib. The fusion protein BCR-ABL produced by a reciprocal translocation between chromosomes 22 and 9 is encoded by sequences from both BCR and ABL. BCR-ABL is endowed with unregulated constitutively active kinase activity, thus stimulates multiple signaling pathways and acts a crucial factor in the pathogenesis of chronic myeloid leukemia. Due to the ability of inhibiting other kinases as well, including PDGF receptor and c-kit, BCR-ABL kinase inhibitors have revealed efficacy in the treatment of other malignancies. Previous studies have demonstrated that the non-mutated endogenous kinase form c-ABL is activated in AD patients' brains and in AD models, suggesting a role in the disease pathogenesis[1]. In mice AD models, treatment with bosutinib or nilotinib resulted in significant reductions in brain Aβ1–42 levels and reduced plaque load, which were associated with improved cognitive performance as measured by Morris water maze tests[2]. Bosutinib and nilotinib could increase parkin levels and restore parkin-Beclin-1 interaction, thus the mechanism of action seems to involve a regulation of autophagic processes

required for amyloid clearance which is impaired in AD brains[2,3]. In addition, kinase inhibitor treatment decreased cytokine and chemokines levels increased by plaque formation[4]. The potential efficacy was tested in a clinical trial (ClinicalTrials.gov: NCT02947893) aimed at testing nilotinib in mild- to-moderate AD. The results showed central amyloid burden reduction in the frontal lobe in the nilotinib group, as well as cerebrospinal fluid Aβ40 decrease at 6 months and 12 months. In addition, hippocampal volume loss was attenuated (−27%) phospho-tau-181 was reduced[5]. On the same line, another study evaluated bosutinib with the aim of investigating longer term tolerability and potential efficacy of tyrosine kinase inhibitors in patients with mild cognitive impairment or dementia (ClinicalTrials.gov: NCT02921477). Imatinib has low brain penetration[2]; however, it is reported to be able to inhibit Aβ production in vitro through inhibition of γ-secretase cleavage[6], as well as by indirectly lowering β-secretase processing of APP[7]. Nevertheless, imatinib treatment reduced plasma and brain Aβ-oligomers levels, plaque load, neuroinflammation, and cognitive deficits in a mouse AD model, suggesting that peripheral mechanisms could contribute to efficacy[8]. However, chronic imatinib treatment in chronic myeloid leukemia patients was unable to lower plasma Aβ1-42 levels[9], thus implying that additional studies are required to clarify this inconsistency.

Crizotinib

Crizotinib inhibits the ALK tyrosine kinase receptor, along with its oncogenic variants, and it is used in lung cancer therapy. In addition to its kinase inhibition properties, crizotinib has been recently recognized as a potent inhibitor of the SH2 domain-containing inositol 5′-phosphatase 2 (SHIP2)[10]. Available evidence indicates SHIP2 as the connecting link between Aβ formation and tau hyperphosphorylation through Fc gamma receptor IIb, since SHIP2 inhibition reduced tau hyperphosphorylation and restored cognitive impairments in a mouse AD model[11]. Overall, these findings support a therapeutic potential against AD.

Afatinib and lapatinib

Afatinib and lapatinib are cancer drugs that inhibit tyrosine kinase receptors belonging to the EGFR family. A potential indication for efficacy in AD derives from evidence for a putative role of the EGFR pathway in suppressing autophagy and the demonstration that its inhibition decreased amyloid-β secretion in vitro and in vivo and improved cognitive functions in AD models[12,13]. In line with these findings, lapatinib reversed memory deficits in a mouse model of cognitive impairment[14], whereas afatinib efficacy in contrasting neuroinflammation suggested a potential efficacy in neurodegenerative diseases[15].

Nintedanib, sorafenib, dabrafenib

Nintedanib and sorafenib are antitumoral agents acting on multiple tyrosine kinase targets which exert their action also by modulating tumor-mediated angiogenesis. Indications supporting repurposing for AD therapy derive from demonstrated efficacy in diminishing neuroinflammatory responses and restoring cognitive abilities in AD mice models[16,17]. Since Raf inhibition has been suggested as a relevant mechanistic target for these responses[18], the Raf inhibitor dabrafenib may also represent a promising drug.

Ruxolitinib and tofacitinib

JAKs inhibitors ruxolitinib and tofacitinib potential efficacy is related to efficacy in dampening excessive inflammatory responses and have therefore already been suggested as objects of repurposing efforts[19,20].

Selumetinib and trametinib

MEK inhibitors selumetinib and trametinib could also possibly act on AD-associated neuroinflammation. Since TREM2 loss of function is one of the strongest known genetic AD risk factors, the discovery that MEK inhibition was able to raise TREM2 cell surface expression and function indicates opportunities for therapeutic intervention with these agents[21].

Pazopanib

Javidnia *et al.* hinted at an anti-inflammatory mechanism for pazopanib-mediated reduction in phosphorylated tau observed in a mouse tauopathy model, although other hypotheses cannot be ruled out[22].

Sunitinib

Sunitinib, which inhibits several tyrosine kinase receptors, has been previously associated to AD therapy with different mechanisms. Indeed, Lee *at al.*[23] identified sunitinib in screenings aimed at identifying molecules able to dissociate Aβ oligomers and plaques to monomers in 5XFAD transgenic mice. However, sunitinib has also been reported to act as an anticholinesterase inhibitor and to attenuate scopolamine-induced impairments of learning and memory in mice similarly to donepezil[24]. Moreover, reversal of AD-associated vascular activation was suggested as mechanism supporting sunitinib-induced improvement in cognitive functions observed in AD mice models[25]. A role for AMP kinase inhibition has also been proposed[26].

Isoprenaline and salbutamol

Isoprenaline is a non-selective β adrenergic receptor agonist and evidence is available for beneficial roles being exerted by β adrenergic signaling through inhibition of tau protein aggregation and reduced neuroinflammation. Inhibition of tau aggregation by isoprenaline has been demonstrated both in vitro and in a mouse model of tauopathy[27]. In agreement with these results, the β2 adrenergic agonist salbutamol has been identified as an effective tau aggregation inhibitor[28]. In addition, treatment with isoprenaline could rescue long-term potentiation from Aβ-induced inhibition through β2 receptors[29], whereas β1 agonist administration improved cognitive impairments and attenuated inflammatory responses in a mouse AD model[30]. Moreover, isoprenaline enhances Aβ42 uptake by microglia, thus enhancing its degradation through insulin-degrading enzyme induction[31]. In contrast, isoprenaline treatment has also been reported to increase amyloid plaque formation through altered γ-secretase trafficking in a mouse AD model[32]. Nevertheless, pro-cognitive and pro-neurogenesis effects have been more recently reported in AD mice after treatment with a β2 adrenergic agonist[33]. Modulation of β adrenergic signaling could contribute to explaining potential efficacy linked to phosphodiesterase inhibitors identified in this study. The clinical potential of β adrenergic receptor agonists in AD was planned to be tested in a

phase 2 clinical trial of formoterol but the study was withdrawn due to the lack of funding (ClinicalTrials.gov: NCT02500784).

Caffeine

Regarding caffeine, after controversial findings, recent data provided further support for a protective effect against cognitive decline and slowing Aβ-amyloid accumulation in coffee consumers, possibly due to caffeine itself[34], and this benefit may extend to other phosphodiesterase inhibitors. Indeed, this class of drugs has also been highlighted in another repurposing effort[35] and clinical trials are ongoing to collect further evidence on their potential efficacy[36,37].

Bromocriptine

Similarly deriving from repurposing studies, the dopamine D2 receptor agonist bromocriptine is being investigated in a randomized controlled trial for AD based on an induced pluripotent stem cells screening for modifiers of Aβ production[38] (ClinicalTrials.gov: NCT04413344). Moreover, immunosuppressant efficacy has been proposed to contribute to bromocriptine efficacy[39].

Methotrexate

Among other potentially interesting drugs extracted in this study, methotrexate is a folic acid antagonist used as antirheumatic agent, which has been associated with a lower risk of dementia among patients that received it as a treatment for chronic inflammatory diseases[40–42]. The potential mechanism of action is suggested to depend on anti-inflammatory activity.

Belinostat

Belinostat is a histone deacetylase inhibitor which acts as an epigenetic regulator; this class is currently being investigated for several indications, including dementia. Specifically, vorinostat and AMX0035 are currently in trials for AD (ClinicalTrials.gov: NCT03056495 and NCT03533257) which are expected to generate useful results[43].

Auranofin

Auranofin mechanism of action is based on the regulation of reduction/oxidation enzymes to keep oxidative stress at low levels and a few data in a mouse AD model support its potentially beneficial effect[44].

Acitretin

Acitretin as a retinoic acid analogue which can be useful in AD due to the ability to regulate ADAM10 promoter and increase its expression, thus diminishing proteolytic processing products of the amyloidogenic pathway[45]. Efficacy has been investigated in a clinical trial (ClinicalTrials.gov: NCT01078168), which showed that acitretin enhances the nonamyloidogenic APP processing in human patients[46].

Doconexent

Doconexent, an ω-3 fatty acid supplement with anti-inflammatory effects proposed as aid to prevent AD based on reported harmful effects of dyslipidemia on AD risk[47]. Overall, clinical

studies seem not to provide support to doconexent preventative effectiveness, although subtle improvements have been observed in specific cognitive abilities and slowed cognitive decline has been reported (ClinicalTrials.gov: NCT04972643[48]; ClinicalTrials.gov: NCT00672685[49]; Chinese Clinical Trial Registry: ChiCTR-IOR-15006058[50]; PMID: 29097166 Netherlands Trial Register NTR1705[51]; PMID: 27716665 ChiCTR-IOR-15006058[52]; ClinicalTrials.gov: NCT00440050).

Everolimus and sirolimus

Everolimus and sirolimus act as mTOR (mammalian target of rapamycin) inhibitors and are used as immunosuppressants. In support of potential efficacy as AD therapeutic agents, recent evidence sustains mTOR role in cognition, plaque and tangle formation, inflammation, oxidative stress, and autophagy, although its utility is hampered by side effects[53].

References

1. Schlatterer, S. D., Acker, C. M. & Davies, P. c-Abl in neurodegenerative disease. *J. Mol. Neurosci.* **45**, 445–452 (2011).
2. Lonskaya, I., Hebron, M. L., Desforges, N. M., Franjie, A. & Moussa, C. E. H. Tyrosine kinase inhibition increases functional parkin-Beclin-1 interaction and enhances amyloid clearance and cognitive performance. *EMBO Mol. Med.* **5**, 1247–1262 (2013).
3. Lonskaya, I., Hebron, M. L., Desforges, N. M., Schachter, J. B. & Moussa, C. E. H. Nilotinib-induced autophagic changes increase endogenous parkin level and ubiquitination, leading to amyloid clearance. *J. Mol. Med. (Berl).* **92**, 373–386 (2014).
4. Lonskaya, I., Hebron, M. L., Selby, S. T., Turner, R. S. & Moussa, C. E. H. Nilotinib and bosutinib modulate pre-plaque alterations of blood immune markers and neuro-inflammation in Alzheimer's disease models. *Neuroscience* **304**, 316–327 (2015).
5. Turner, R. S. *et al.* Nilotinib Effects on Safety, Tolerability, and Biomarkers in Alzheimer's Disease. *Ann. Neurol.* **88**, 183–194 (2020).
6. Netzer, W. J. *et al.* Gleevec inhibits beta-amyloid production but not Notch cleavage. *Proc. Natl. Acad. Sci. U. S. A.* **100**, 12444–12449 (2003).
7. Netzer, W. J. *et al.* Gleevec shifts APP processing from a β-cleavage to a nonamyloidogenic cleavage. *Proc. Natl. Acad. Sci. U. S. A.* **114**, 1389–1394 (2017).
8. Estrada, L. D. *et al.* Reduction of Blood Amyloid-β Oligomers in Alzheimer's Disease Transgenic Mice by c-Abl Kinase Inhibition. *J. Alzheimers. Dis.* **54**, 1193–1205 (2016).
9. Olsson, B. *et al.* Imatinib treatment and Aβ42 in humans. *Alzheimers. Dement.* **10**, S374–S380 (2014).
10. Lim, J. W. *et al.* Identification of crizotinib derivatives as potent SHIP2 inhibitors for the treatment of Alzheimer's disease. *Eur. J. Med. Chem.* **157**, 405–422 (2018).
11. Kam, T. I. *et al.* FcγRIIb-SHIP2 axis links Aβ to tau pathology by disrupting phosphoinositide metabolism in Alzheimer's disease model. *Elife* **5**, e18691 (2016).

12. Wang, B. J. *et al.* ErbB2 regulates autophagic flux to modulate the proteostasis of APP-CTFs in Alzheimer's disease. *Proc. Natl. Acad. Sci. U. S. A.* **114**, E3129–E3138 (2017).

13. Tavassoly, O., Sato, T. & Tavassoly, I. Inhibition of Brain Epidermal Growth Factor Receptor Activation: A Novel Target in Neurodegenerative Diseases and Brain Injuries. *Mol. Pharmacol.* **98**, 13–22 (2020).

14. Mansour, H. M., Fawzy, H. M., El-Khatib, A. S. & Khattab, M. M. Lapatinib ditosylate rescues memory impairment in D-galactose/ovariectomized rats: Potential repositioning of an anti-cancer drug for the treatment of Alzheimer's disease. *Exp. Neurol.* **341**, 113697 (2021).

15. Chen, Y. J. *et al.* Anti-inflammatory effect of afatinib (an EGFR-TKI) on OGD-induced neuroinflammation. *Sci. Rep.* **9**, 2516 (2019).

16. Kim, J., Park, J. H., Park, S. K. & Hoe, H. S. Sorafenib Modulates the LPS- and Aβ-Induced Neuroinflammatory Response in Cells, Wild-Type Mice, and 5xFAD Mice. *Front. Immunol.* **12**, 684344 (2021).

17. Echeverria, V. *et al.* Sorafenib inhibits nuclear factor kappa B, decreases inducible nitric oxide synthase and cyclooxygenase-2 expression, and restores working memory in APPswe mice. *Neuroscience* **162**, 1220–1231 (2009).

18. Burgess, S. & Echeverria, V. Raf inhibitors as therapeutic agents against neurodegenerative diseases. *CNS Neurol. Disord. Drug Targets* **9**, 120–127 (2010).

19. Hasselbalch, H. C. *et al.* Myeloproliferative blood cancers as a human neuroinflammation model for development of Alzheimer's disease: evidences and perspectives. *J. Neuroinflammation* **17**, 248 (2020).

20. Desai, R. J. *et al.* Targeting abnormal metabolism in Alzheimer's disease: The Drug Repurposing for Effective Alzheimer's Medicines (DREAM) study. *Alzheimer's Dement. (New York, N. Y.)* **6**, e12095 (2020).

21. Schapansky, J. *et al.* MEK1/2 activity modulates TREM2 cell surface recruitment. *J. Biol. Chem.* **296**, 100218 (2021).

22. Javidnia, M., Hebron, M. L., Xin, Y., Kinney, N. G. & Moussa, C. E. H. Pazopanib Reduces Phosphorylated Tau Levels and Alters Astrocytes in a Mouse Model of Tauopathy. *J. Alzheimers. Dis.* **60**, 461–481 (2017).

23. Lee, J. C. *et al.* Discovery of Chemicals to Either Clear or Indicate Amyloid Aggregates by Targeting Memory-Impairing Anti-Parallel Aβ Dimers. *Angew. Chem. Int. Ed. Engl.* **59**, 11491–11500 (2020).

24. Huang, L. *et al.* Sunitinib, a Clinically Used Anticancer Drug, Is a Potent AChE Inhibitor and Attenuates Cognitive Impairments in Mice. *ACS Chem. Neurosci.* **7**, 1047–1056 (2016).

25. Grammas, P. *et al.* A new paradigm for the treatment of Alzheimer's disease: targeting vascular activation. *J. Alzheimers. Dis.* **40**, 619–630 (2014).

26. Son, S. M., Jung, E. S., Shin, H. J., Byun, J. & Mook-Jung, I. Aβ-induced formation of autophagosomes is mediated by RAGE-CaMKKβ-AMPK signaling. *Neurobiol. Aging* **33**, 1006.e11-1006.e23 (2012).

27. Soeda, Y. *et al.* Toxic tau oligomer formation blocked by capping of cysteine residues with 1,2-dihydroxybenzene groups. *Nat. Commun.* **6**, 10216 (2015).

28. Townsend, D. J. *et al.* Circular Dichroism Spectroscopy Identifies the β-Adrenoceptor Agonist Salbutamol As a Direct Inhibitor of Tau Filament Formation in Vitro. *ACS Chem. Neurosci.* **11**, 2104–2116 (2020).

29. Wang, Q. wen, Rowan, M. J. & Anwyl, R. Inhibition of LTP by beta-amyloid is prevented by activation of beta2 adrenoceptors and stimulation of the cAMP/PKA signalling pathway. *Neurobiol. Aging* **30**, 1608–1613 (2009).

30. Ardestani, P. M. *et al.* Modulation of neuroinflammation and pathology in the 5XFAD mouse model of Alzheimer's disease using a biased and selective beta-1 adrenergic receptor partial agonist. *Neuropharmacology* **116**, 371–386 (2017).

31. Kong, Y., Ruan, L., Qian, L., Liu, X. & Le, Y. Norepinephrine promotes microglia to uptake and degrade amyloid beta peptide through upregulation of mouse formyl peptide receptor 2 and induction of insulin-degrading enzyme. *J. Neurosci.* **30**, 11848–11857 (2010).

32. Ni, Y. *et al.* Activation of beta2-adrenergic receptor stimulates gamma-secretase activity and accelerates amyloid plaque formation. *Nat. Med.* **12**, 1390–1396 (2006).

33. Chai, G. S., Wang, Y. Y., Yasheng, A. & Zhao, P. Beta 2-adrenergic receptor activation enhances neurogenesis in Alzheimer's disease mice. *Neural Regen. Res.* **11**, 1617–1624 (2016).

34. Gardener, S. L. *et al.* Higher Coffee Consumption Is Associated With Slower Cognitive Decline and Less Cerebral Aβ-Amyloid Accumulation Over 126 Months: Data From the Australian Imaging, Biomarkers, and Lifestyle Study. *Front. Aging Neurosci.* **13**, 744872 (2021).

35. Fang, J. *et al.* Endophenotype-based in silico network medicine discovery combined with insurance record data mining identifies sildenafil as a candidate drug for Alzheimer's disease. *Nat. aging* **1**, 1175–1188 (2021).

36. Sanders, O. & Rajagopal, L. Phosphodiesterase Inhibitors for Alzheimer's Disease: A Systematic Review of Clinical Trials and Epidemiology with a Mechanistic Rationale. *J. Alzheimer's Dis. reports* **4**, 185–215 (2020).

37. Sanders, O. Sildenafil for the Treatment of Alzheimer's Disease: A Systematic Review. *J. Alzheimer's Dis. reports* **4**, 91–106 (2020).

38. Kondo, T. *et al.* Repurposing bromocriptine for Aβ metabolism in Alzheimer's disease (REBRAnD) study: randomised placebo-controlled double-blind comparative trial and open-label extension trial to investigate the safety and efficacy of bromocriptine in Alzheimer's disease with presenilin 1 (PSEN1) mutations. *BMJ Open* **11**, e051343 (2021).

39. Tucker Edmister, S. *et al.* Novel use of FDA-approved drugs identified by cluster analysis of behavioral profiles. *Sci. Rep.* **12**, 6120 (2022).

40. Newby, D. *et al.* Methotrexate and relative risk of dementia amongst patients with rheumatoid arthritis: a multi-national multi-database case-control study. *Alzheimers. Res. Ther.* **12**, 38 (2020).

41. Zhou, M., Xu, R., Kaelber, D. C. & Gurney, M. E. Tumor Necrosis Factor (TNF) blocking agents are associated with lower risk for Alzheimer’s disease in patients with rheumatoid arthritis and psoriasis. *PLoS One* **15**, e0229819 (2020).

42. Judge, A. *et al.* Protective effect of antirheumatic drugs on dementia in rheumatoid arthritis patients. *Alzheimer’s Dement. (New York, N. Y.)* **3**, 612–621 (2017).

43. Bondarev, A. D. *et al.* Recent developments of HDAC inhibitors: Emerging indications and novel molecules. *Br. J. Clin. Pharmacol.* **87**, 4577–4597 (2021).

44. Upīte, J., Kadish, I., van Groen, T. & Jansone, B. Subchronic administration of auranofin reduced amyloid-β plaque pathology in a transgenic APP NL-G-F/NL-G-F mouse model. *Brain Res.* **1746**, 147022 (2020).

45. Fahrenholz, F., Tippmann, F. & Endres, K. Retinoids as a perspective in treatment of Alzheimer’s disease. *Neurodegener. Dis.* **7**, 190–192 (2010).

46. Endres, K. *et al.* Increased CSF APPs-α levels in patients with Alzheimer disease treated with acitretin. *Neurology* **83**, 1930–1935 (2014).

47. Reitz, C. Dyslipidemia and the risk of Alzheimer’s disease. *Curr. Atheroscler. Rep.* **15**, 307 (2013).

48. Lin, P. Y. *et al.* Omega-3 fatty acids and blood-based biomarkers in Alzheimer’s disease and mild cognitive impairment: A randomized placebo-controlled trial. *Brain. Behav. Immun.* **99**, 289–298 (2022).

49. Andrieu, S. *et al.* Effect of long-term omega 3 polyunsaturated fatty acid supplementation with or without multidomain intervention on cognitive function in elderly adults with memory complaints (MAPT): a randomised, placebo-controlled trial. *Lancet. Neurol.* **16**, 377–389 (2017).

50. Zhang, Y. P., Lou, Y., Hu, J., Miao, R. & Ma, F. DHA supplementation improves cognitive function via enhancing Aβ-mediated autophagy in Chinese elderly with mild cognitive impairment: a randomised placebo-controlled trial. *J. Neurol. Neurosurg. Psychiatry* **89**, 382–388 (2018).

51. Soininen, H. *et al.* 24-month intervention with a specific multinutrient in people with prodromal Alzheimer’s disease (LipiDiDiet): a randomised, double-blind, controlled trial. *Lancet. Neurol.* **16**, 965–975 (2017).

52. Zhang, Y. P., Miao, R., Li, Q., Wu, T. & Ma, F. Effects of DHA Supplementation on Hippocampal Volume and Cognitive Function in Older Adults with Mild Cognitive Impairment: A 12-Month Randomized, Double-Blind, Placebo-Controlled Trial. *J. Alzheimers. Dis.* **55**, 497–507 (2017).

53. Rapaka, D., Bitra, V. R., Challa, S. R. & Adiukwu, P. C. mTOR signaling as a molecular target for the alleviation of Alzheimer's disease pathogenesis. *Neurochem. Int.* **155**, 105311 (2022).
