# Supplementary material for: Single-cell-led drug repurposing for Alzheimer’s disease
Source: Sci Rep. 2023 Jan 5;13:222. doi: 10.1038/s41598-023-27420-x (PMC9816180; doi:10.1038/s41598-023-27420-x)
Supplement: Supplementary file 8 — Supplementary Information 8. [file 41598_2023_27420_MOESM8_ESM.docx]

**Supplementary Table 1. List of disease genes and supporting evidence information for each cell type.**

**Supplementary Table 2. List of network modules significantly enriched in disease genes.**

**Supplementary Table 3. Results of ligand-receptor analysis.**

**Supplementary Table 4. Results of Reactome pathway enrichment analysis for the core AD network modules.**

**Supplementary Table 5. Open Targets annotation of the targets identified by the drug repurposing pipeline.**

**Supplementary Table 6. Results of Reactome pathway enrichment analysis for the targets of AD drug repurposing candidates.**
